# The prognostic and predictive value of platelet parameters in diabetic and nondiabetic patients with sudden sensorineural hearing loss

**DOI:** 10.1515/biol-2020-0083

**Published:** 2020-12-04

**Authors:** İbrahim Özcan, İbrahim Hira, Altan Kaya, Mehmet Yaşar, Murat Doğan, Cemil Mutlu

**Affiliations:** Kayseri City Hospital, ENT Clinic. Şeker Mah. Molu Cad. Kocasinan, Kayseri, Turkey

**Keywords:** sudden hearing loss, platelet parameters, prognosis, diabetes mellitus

## Abstract

**Background:**

We aimed to evaluate the association between mean platelet volume, platelet distribution width (PDW), platelet count (PC) and plateletcrit (PCT), and the presence of sudden sensorineural hearing loss (SSNHL) and treatment response. In the literature, there is no study that investigates the platelet functions in diabetic patients with SSNHL.

**Methods:**

The patients were retrospectively assigned into Group 1 (68 diabetic patients with SSNHL), Group 2 (63 nondiabetic patients with SSNHL) and Group 3 (64 healthy controls).

**Results:**

PC was not significantly different between the groups (*p* > 0.05). MPV, PDW and PCT values were significantly higher in Group 1 as compared to Groups 2 and 3 (*p* < 0.05). Platelet parameters were not significantly different between the patients who were responsive and nonresponsive to the treatment. Therefore, the platelet parameters did not affect prognosis significantly in this study samples (*p* > 0.05).

**Conclusions:**

This study showed that platelet parameters did not have a significant effect as a prognostic and predictive value in diabetic and nondiabetic patients with SSNHL. Further studies with more homogenous and larger study groups investigating the platelet parameters are needed to demonstrate microvascular damage and vascular alterations induced by diabetes mellitus.

## 1 Introduction

Sudden sensorineural hearing loss (SSNHL) is defined as a 30 dB or more sensorineural hearing loss that occurs within 72 h at least in three consecutive frequencies. The prevalence of the disease is reported to be 5–20/100,000. The etiological factors can be determined in 10–15% of the cases, approximately [1,2,3]. Although it may occur at any age, SSNHL is rarely seen in the childhood with a worse prognosis and prevalent between ages of 50 and 60 with no gender difference. A number of factors regarding the disease such as etiology, treatment protocols and prognosis have not been fully elucidated to date. Infectious, vascular, cardiologic, immunologic, hematologic, genetic and ototoxic factors, stress, perinatal complications, rheumatologic diseases, diabetes mellitus (DM), tumors and cardiovascular surgeries are claimed as etiological factors [3,4,5,6,7]. Also, studies reporting the increased frequencies of SSNHL in pathological conditions including sickle cell anemia, macroglobulinemia, Burger, cardiovascular and connective tissue diseases exist in the literature [5,6]. Neutrophil, lymphocyte and platelet values derived from routine hemogram analyses and their proportions were determined as prognostic and predictive indicators in several studies without any widely accepted precise parameters [8,9,10,11,12,13,14,15]. In SSNHL, tinnitus, dizziness, nausea and vomiting, aural fullness and findings of the upper airway infection can also be seen along with the hearing loss [4,16].

Mean platelet volume (MPV) is an indicator that addresses the size, function and production rate of platelets. The platelets with bigger sizes are more active and prone to aggregate [12,17]. A number of studies reported a correlation between MPV values and cardiovascular diseases, stroke, thrombosis and inflammation [10,13,17]. Platelet distribution width (PDW), platelet count (PC) and plateletcrit (PCT) also constitute other important parameters. PDW characteristically reflects the discrepancies in platelet volumes.

In the present study, we aimed to evaluate the association between SSNHL in diabetic and nondiabetic patients, and MPV, PDW, PC and PCT values with the determination of predictive and prognostic values of these parameters. Platelet values were reported in different studies with contradictory results in the literature [9,10,11,12,13,14,15]. However, studies concerning their prognostic value are still limited with the absence of a study focused on platelet values and DM.

## 2 Materials and methods

### 2.1 Patients

The present study involved a total of 131 patients, including 68 diabetic and 63 nondiabetic patients who had been diagnosed with SSNHL at Kayseri Training and Research Hospital between March 2016 and July 2018. Age- and sex-matched 64 participants served as the control group. SSNHL was defined as a hearing loss of 30 dB or greater at least in three consecutive frequencies, over a period of 72 h or less [1]. All patients received 1 mg/kg systemic methylprednisolone (Mustafa Nevzat İlaç Sanayi A.Ş., Istanbul, Turkey) with a tapering dose of 20 mg every 3 days. Follow-up and regulation of blood glucose levels were performed in all patients. Oral lansoprazol (Nobel İlaç Sanayi A.Ş., Istanbul, Turkey) 30 mg/day was prescribed for gastric mucosal protection. Intratympanic dexamethasone (ITS; Deva Holding A.Ş., Istanbul, Turkey) and/or hyperbaric oxygen therapy (HBOT) were combined with systemic steroid treatment as salvage therapy. Five doses of ITS were applied every other day. Patients received ten sessions of HBOT composed of 2.5 ATA for 120 min per day. Patients who were benefited from HBOT according to audiometric findings received additional ten sessions of HBOT.

The inclusion criteria for the study were as follows: 18–65 age, initiation of therapy in the first 7 days and no treatment before the application in our clinic.

Patients with retrocochlear pathology, ototoxicity, perilymphatic fistula and acoustic trauma, history of previous SSNHL and ear surgery, presence of ear disease (tympanic membrane perforation, chronic otitis media, etc.), autoimmune disease, hyperlipidemia, chronic renal failure, cardiovascular disease and newly diagnosed DM were excluded from the study. DM was diagnosed when HbA1c was ≥6.5%.

In this study, diabetic patients and nondiabetic patients with SSNHL were assigned into Group 1 and Group 2, respectively, whereas healthy controls were included in Group 3.


**Informed consent:** Informed consent has been obtained from all individuals included in this study.
**Ethical approval:** The research related to human use has been complied with all the relevant national regulations, institutional policies and in accordance with the tenets of the Helsinki Declaration and has been approved by the authors’ institutional review board or equivalent committee.

### 2.2 Audiometric evaluation

The diagnosis of SSNHL was performed according to a complete otorhinolaryngological examination and pure tone audiometry (PTA). Pure-tone thresholds of 250–8,000 Hz frequencies and speech discrimination scores were evaluated in PTA. Patients were classified according to the Siegel criteria in agreement with the initial and 3rd-month hearing thresholds. The patients were categorized into four types according to Siegel’s criteria based on their hearing thresholds at the beginning and at the end of the 3rd month (Table 1).

**Table 1:** Study groups according to Siegel’s criteria

Type	Hearing recovery
I. Complete recovery (Type 1)	Final hearing better than 25 dB
II. Partial recovery (Type 2)	More than 15 dB gain, final hearing 25–45 dB
III. Slight recovery (Type 3)	More than 15 dB gain, final hearing poorer than 45 dB
IV. No improvement (Type 4)	Less than 15 dB gain

Siegel Types 1, 2 and 3 were deemed as responsive for treatment, while Siegel Type 4 was regarded as unresponsive.

### 2.3 Biochemical and hematological evaluation

Before the commencement of treatment, all patients were examined in terms of full blood count and hemostasis panel. Blood samples were collected in EDTA tubes in order to analyze MPV, PDW, PCT and PC values.

### 2.4 Statistical evaluation

Data were analyzed using the SPSS software v22 (SPSS Inc, Chicago, IL, USA). Normality of data distribution was analyzed using the Kolmogorov–Smirnov test. Mann–Whitney *U* and Kruskal–Wallis tests were used for quantitative comparisons, whereas Wilcoxon’s signed-rank and Pearson’s chi-square tests were performed for comparisons between the groups. Significance was considered for *p* < 0.05 for all comparisons.

## 3 Results

**Figure 1:**
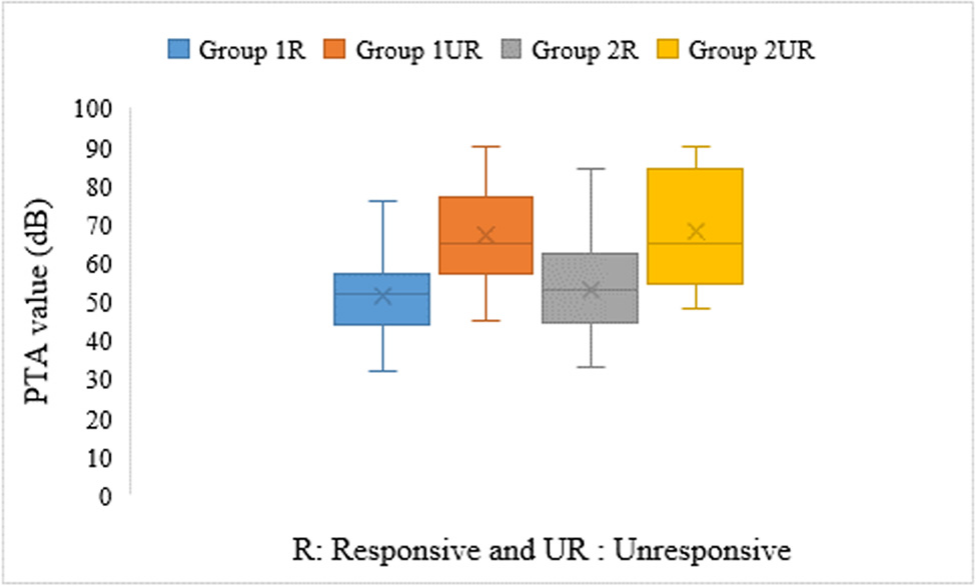
Distribution of pretreatment PTA values in the groups.

Groups 1, 2 and 3 consisted of 68, 63 and 64 patients, respectively. There was no significant difference between the groups in terms of age and sex distributions (Table 2). The pretreatment average PTAs were 55.47 ± 24.32 and 53.59 ± 22.05 in Group 1 and Group 2, respectively, and there were no statistically significant differences between these values. But, in both groups, the values were significantly lower in responsive patients than in unresponsive patients (*p* = 0.036; Figure 1).

**Table 2:** Demographic and laboratory data of study groups

		Group 1	Group 2	Group 3	*p*
Number of subjects		68	63	64	>0.05
Age		45.28 ± 16.09	44.31 ± 14.32	39.26 ± 18.39	>0.05
Gender	Female	36	35	36	>0.05
	Male	32	28	28	
MPV (fL)		10.81 ± 2.09*	10.01 ± 1.12*^+^	9.93 ± 0.71^+^	*0.037, ^+^0.104
PDW (%)		15.31 ± 1.62*	14.02 ± 1.82*^+^	13.35 ± 2.09^+^	*0.046, ^+^0.237
PCT (%)		0.31 ± 0.08*	0.30 ± 0.05*^+^	0.28 ± 0.04^+^	*0.032, ^+^0.087
PC (10^3^/µL)		278 ± 82	272 ± 62	268 ± 74	>0.05

MPV: mean platelet volume, PDW: platelet distribution width, PCT: plateletcrit, PC: platelet count. The (*) symbol indicates the *p* value for the evaluation between Group 1 and Group 2, and the (+) symbol for the evaluation between Group 2 and Group 3.

The comparisons of MPV, PDW, PCT and PC values between the groups are shown in Table 2. MPV, PDW and PCT values were significantly higher in Group 1 as compared to Groups 2 and 3, while there was no significant difference between Groups 2 and 3. Also, no significant difference was found between the groups in terms of PC. There was no significant difference in terms of platelet parameters between responsive and unresponsive patients to the treatment in Groups 1 and 2 (Table 3).

**Table 3:** Effect of platelet parameters on prognosis in diabetic and nondiabetic patients

		Group 1		Group 2		*p*
		Responsive	Unresponsive	Responsive	Unresponsive	
Number of subjects		51	17	54	9	<0.05
Age		44.28 ± 13.09	45.88 ± 17.29	42.28 ± 10.34	45.37 ± 12.98	>0.05
Gender	Female	22	14	24	11	<0.05
	Male	17	15	19	9	
MPV (fL)		10.21 ± 1.49	10.88 ± 1.82	9.93 ± 1.42	10.08 ± 1.02	>0.05
PDW (%)		14.81 ± 2.62	15.38 ± 1.47	14.02 ± 1.32	14.12 ± 1.58	>0.05
PCT (%)		0.30 ± 0.18	0.33 ± 0.01	0.29 ± 0.09	0.30 ± 0.38	>0.05
PC (10^3^/µL)		268 ± 58	283 ± 62	263 ± 51	271 ± 29	>0.05

MPV: mean platelet volume, PDW: platelet distribution width, PCT: plateletcrit, PC: platelet count. The *p* value indicates the assessment between the responding and nonresponding patient groups in Group 1 and Group 2.

## 4 Discussion

The etiology, pathogenesis and treatment modalities of SSNHL have not yet been fully understood. There are several studies concerning SSNHL in the literature. Due to the higher prevalence of SSNHL among the patients with cardiovascular disease and thromboembolic risk factors, vascular occlusion theory is widely accepted. The absence of collateral blood supply supports the theory. SSNHL is also prevalent in patients with DM and hypercholesterolemia who have higher atherosclerotic risk factors [6,18]. Platelets play an important role in the presence of atherothrombosis and interact with leukocytes in the damaged endothelium resulting in thrombus formation. The process of inflammation triggers the presence of thrombus formation and vice versa. In particular, platelets with higher volumes are more prone to aggregate with platelet agonists such as ADP, collagen and adrenalin. These platelets secrete more prothrombotic and vasoactive factors including thromboxane A2, serotonin and ATP; therefore, they have a more inflammatory characteristic [19]. During the inflammatory process, the release of integrins located over the platelets such as p-selectin raises and causes increased adhesion and thrombotic environment. Accordingly, high MPV levels were detected in several vascular and inflammatory events such as DM, myocardial infarction, stroke and sepsis [20,21]. Due to the presence of insulin resistance in DM, platelet turnover accelerates, the platelet life span shortens and platelet parameters may interrupt including MPV and PDW levels. MPV values were found to be higher in patients with retinopathy and microalbuminuria [22]. Therefore, in the present study, we constituted an extra group composed of SSNHL and DM in order to reveal the alteration of platelet parameters in detail. Platelet parameters derived from the complete blood count were studied in several pathological conditions including cardiovascular, inflammatory and oncological diseases due to being a cheap and routine laboratory examination and revealed contradictory results in these studies [17,18,19,20,21,22,23,24,25].

The effect of platelet parameters on the prognosis of SSNHL has not been extensively investigated in the literature. Also, to our knowledge, there is no study in the literature that has investigated platelet parameters in diabetic patients with SSNHL. On the other hand, the studies focusing on the predictive value of parameters have revealed conflicting results. Seo et al. [14] reported that PC had a predictive value but had no prognostic significance. In another study, PC was also found to have no predictive value [15]. In the present study, PC was also found to have no significant predictive and prognostic values.

In the study by Ulu et al. [10] conducted in 2013, the authors concluded that the MPV and PDW values were significantly higher in the study group when compared to the control group, whereas PC was lower. However, results contradictory to Ulu et al. exist in the literature [9,12]. In the present study, MPV, PDW and PCT values were significantly higher in Group 1 as compared to Groups 2 and 3, whereas there was no significant difference between Groups 2 and 3 (Table 1). Ji et al. [26] reported a meta-analysis and demonstrated that there was no significant difference among the MPV values and between controls. Sun et al. [27] reported an association between MPV values and poor prognosis.

The prevalence of DM was reported higher among the patients with SSNHL [28]. Aimoni et al. [6] found that the prevalence of DM was 15.6% among the patients with SSNHL, whereas it was 8.5% among the control group. This result supported that DM has induced microangiopathy. Weng et al. [29] reported that hearing loss in the contralateral ear and the profound-type hearing loss in the lesion ear were commonly noted in diabetic patients with SSNHL. Also, the authors determined that diabetic patients with SSNHL had a poor prognosis that might be attributed to the microvascular damage of the inner ear. On the contrary, Seo et al. [30] found that there is no difference between diabetic and nondiabetic SSNHL patients on recovery when matched with sex, age and initial hearing level, so DM itself is not a poor prognostic indicator for SSNHL. But, in the present study, recovery values were significantly lower in diabetics (Group 1) as compared to nondiabetics (Group 2).

In the present study, we aimed to investigate the prognostic value of platelet parameters (MPV, PDW and PCT) in diabetic patients with SSNHL and also evaluate whether it is an indirect finding of microvascular damage in DM [23,24,25].

In the present study, we found that the values of platelet parameters (MPV, PDW and PCT) were significantly higher in Group 1 than those of Group 2. However, the difference was not significant between the patients who were responsive and nonresponsive to the treatment protocol in Groups 1 and 2. Therefore, we have concluded that these parameters did not have a prognostic value.

The present study has some limitations. In particular, the prognostic value of platelet parameters and microvascular damage may be reflected more objectively if the additional variables regarding the duration and treatment protocols of DM and the status of the blood regulation were included in the present study. Larger studies with more homogenous patient groups may be useful for elucidating the relationship between DM and platelet parameters.

## 5 Conclusion

In the present study, we have concluded that the platelet parameters (MPV, PDW, PCT and PC) alone had no prognostic and predictive values in the diabetic and nondiabetic patients with SSNHL. Further studies are required for evaluating the microvascular damage and vascular alterations induced by DM.

## References

[1] Byl FM. Seventy-six cases of presumed sudden hearing loss occurring in 1973: prognosis and incidence. Laryngoscope. 1977;87(5 Pt 1):817–25. 10.1002/lary.5540870515 850455

[2] Dallan I, Bruschini L, Nacci A, Bruschini P, Traino C, Rognini F, et al. Transtympanic steroids as a salvage therapy in sudden hearing loss: preliminary results. ORL J Otorhinolaryngol Relat Spec. 2006;68(5):247–52. 10.1159/000093093 16679810

[3] De Felice C, De Capua B, Tassi R, Mencattini G, Passàli D. Non-functioning posterior communicating arteries of circle of Willis in idiopathic sudden hearing loss. Lancet. 2000;356(7):1237–8. 10.1016/S0140-6736(00)02790-2 11072945

[4] Schreiber BE, Agrup C, Haskard DO, Luxon LM. Sudden sensorineural hearing loss. Lancet. 2010;375:1203–11. 10.1016/S0140-6736(09)62071-7 20362815

[5] Penido NO, Cruz OL, Zanoni A, Inoue DP. Classification and hearing evolution of patients with sudden sensorineural hearing loss. Braz J Med Biol Res. 2009;42(8):712–6. 10.1590/s0100-879x2009000800004 19649397

[6] Aimoni C, Bianchini C, Borin M, Ciorba A, Fellin R, Martini A, et al. Diabetes, cardiovascular risk factors and idiopathic sudden sensorineural hearing loss: a case-control study. Audiol Neurootol. 2010;15(2):111–5. 10.1159/000231636 19657186

[7] Hato N, Hyodo J, Takeda S, Takagi D, Okada M, Hakuba N, et al. Local hypothermia in the treatment of idiopathic sudden sensorineural hearing loss. Auris Nasus Larynx. 2010;37(5):626–30. 10.1016/j.anl.2010.01.008 20167446

[8] Masuda M, Kanzaki J. Cause of idiopathic sudden sensorineural hearing loss: the stress response theory. World J Otorhinolaryngol. 2013;28(3):42–57.

[9] Mirvakili A, Dadgarnia MH, Baradaranfar MH, Atighechi S, Zand V, Ansari A. Role of platelet parameters on sudden sensorineural hearing loss: a case-control study in Iran. PLoS One. 2016;11(2):e0148149. 10.1371/journal.pone.0148149 PMC4734775 26829393

[10] Ulu S, Ulu MS, Ahsen A, Yucedag F, Aycicek A, Celik S. Increased levels of mean platelet volume: a possible relationship with idiopathic sudden hearing loss. Eur Arch Otorhinolaryngol. 2013;270(11):2875–8. 10.1007/s00405-013-2348-9 23341093

[11] Balta S, Demirkol S, Yildizoglu U, Arslan Z, Unlu M, Celik T. Other inflammatory markers ought to be kept in mind when assessing the mean platelet volume in clinical practice. Eur Arch Otorhinolaryngol. 2013;270(8):2373–4. 10.1007/s00405-013-2522-0 23616142

[12] Karli R, Alacam H, Unal R, Kucuk H, Aksoy A, Ayhan E. Mean platelet volume: is it a predictive parameter in the diagnosis of sudden sensorineural hearing loss? Indian J Otolaryngol Head Neck Surg. 2013;65(4):350–3. 10.1007/s12070-013-0648-4 PMC3851518 24427597

[13] Blaha M, Kostal M, Drsata J, Chrobok V, Lanska M, Zak P. Does mean platelet volume really increase in sudden sensorineural hearing loss. Eur Arch Otorhinolaryngol. 2015;272(9):2575–8. 10.1007/s00405-014-3384-9 25451541

[14] Seo YJ, Jeong JH, Choi JY, Moon IS. Neutrophil-to-lymphocyte ratio and platelet-to-lymphocyte ratio: novel markers for diagnosis and prognosis in patients with idiopathic sudden sensorineural hearing loss. Dis Markers. 2014;2014:702807. 10.1155/2014/702807 PMC4033535 24891755

[15] Kum RO, Ozcan M, Baklaci D, Yurtsever Kum N, Yilmaz YF, Unal A, et al. Investigation of neutrophil-to-lymphocyte ratio and mean platelet volume in sudden hearing loss. Braz J Otorhinolaryngol. 2015;81(6):636–41. 10.1016/j.bjorl.2015.08.009 PMC9442714 26480902

[16] Stachler RJ, Chandrasekhar SS, Archer SM, Rosenfeld RM, Schwartz SR, Barrs DM, et al. Clinical practice guideline: sudden hearing loss. Otolaryngol Head Neck Surg. 2012;146(3 Suppl):S1–35. 10.1177/0194599812436449 22383545

[17] Bodur S, Gun I, Babayigit MA. The significance of mean platelet volume on diagnosis and management of adenomyosis. Med Glas (Zenica). 2013;10(1):59–62. 23348163

[18] Leys D, Deplanque D, Mounier-Vehier C, Mackowiak-Cordoliani MA, Lucas C, Bordet R. Stroke prevention: management of modifiable vascular risk factors. J Neurol. 2002;249:507–17. 10.1007/s004150200057 12021938

[19] Bath PM, Butterworth RJ. Platelet size: measurement, physiology and vascular disease. Blood Coagul Fibrinol. 1996;7(2):157–61. 8735807

[20] Becchi C, Al Malyan M, Fabbri LP, Marsili M, Boddi V, Boncinelli S. Mean platelet volüme trend in sepsis: is it a useful parameter? Minerva Anestesiol. 2006;72(9):749–56. 16871155

[21] Shah AR, Chaudhari SN, Shah MH. Role of platelet parameters in diagnosis various clinical conditions. Natl J Med Res. 2013;3(2):162–5.

[22] Papanas N, Symeonidis G, Maltezos E, Mavridis G, Karavageli E, Vosnakidis T. Mean platelet volume in patients with type 2 diabetes mellitus. Platelets. 2004;15(8):475–8. 10.1080/0953710042000267707 15763888

[23] Fan Z, Pan J, Zhang Y, Wang Z, Zhu M, Yang B, et al. Mean platelet volume and platelet distribution width as markers in the diagnosis of acute gangrenous appendicitis. Dis Markers. 2015;2015:542013. 10.1155/2015/542013 PMC4673334 26688600

[24] Liu R, Gao F, Huo J, Yi Q. Study on the relationship between mean platelet volume and platelet distribution width with coronary artery lesion in children with Kawasaki disease. Platelets. 2012;23(1):11–6. 10.3109/09537104.2011.586073 21675937

[25] Fan Z, Zhuang C. The relationship of mean platelet volume/platelet distribution width and duodenal ulcer perforation. Ann Clin Lab Sci. 2017;47(2):166–70. 28442518

[26] Ji S, Chen X, Shi H, Zhang B, Yao S, Deng S, et al. Relationship between platelet parameters and sudden sensorineural hearing loss: a systematic review and meta-analysis. Biosci Rep. 2018;38(6):BSR20181183. 10.1042/BSR20181183 PMC6239278 30232233

[27] Sun Y, Guo Y, Wang H, Chen Z, Wu Y, Shi H, et al. Differences in platelet-related parameters among patients with audiographically distinct sudden sensorineural hearing loss: a retrospective study. Med (Baltim). 2017;96(36):e7877. 10.1097/MD.0000000000007877 PMC6392555 28885341

[28] Haremza C, Klopp-Dutote N, Strunski V, Page C. Evaluation of cardiovascular risks and recovery of idiopathic sudden sensorineural hearing loss in hospitalised patients: comparison between complete and partial sudden sensorineural hearing loss. J Laryngol Otol. 2017;131(10):919–24. 10.1017/S0022215117001736 28807070

[29] Weng SF, Chen YS, Hsu CJ, Tseng FY. Clinical features of sudden sensorineural hearing loss in diabetic patients. Laryngoscope. 2005;115:1676–80. 10.1097/01.mlg.0000184790.91675.e3 16148716

[30] Seo HW, Chung JH, Byun H, Jeong JH, Lee SH. Effect of diabetes on the prognosis of sudden sensorineural hearing loss: propensity score matching analysis. Otolaryngol Head Neck Surg. 2020;162(3):346–52. 10.1177/0194599820901359 31959036

